# Differential Uptake and Release of Female Genital Secretions Components and HPV DNA by Veil, Swab, and Vaginal Tampon

**DOI:** 10.3390/diagnostics16030380

**Published:** 2026-01-23

**Authors:** Ralph-Sydney Mboumba Bouassa, Jonathan Muwonga Tukisadila, Laurent Belec

**Affiliations:** 1 Institut du Savoir Montfort, Montfort Hospital, Ottawa, ON K1K 0T2, Canada; ralphsmbouassa@montfort.on.ca; 2 Department of Family Medicine, Faculty of Medicine, University of Ottawa, Ottawa, ON K1N 6S1, Canada; 3 École Doctorale Régionale D’Afrique Centrale en Infectiologie Tropicale, Franceville 876, Gabon; jmmuwonga@gmail.com; 4 Laboratoire de Biologie Clinique des Cliniques Universitaires de Kinshasa, Kinshasa 123, Democratic Republic of the Congo; 5 Laboratory of Virology, Hôpital Européen Georges Pompidou, Assistance Publique-Hôpitaux de Paris (AP-HP), 75015 Paris, France; 6 Faculté de Médecine Paris Descartes, Université Paris Cité, 75006 Paris, France

**Keywords:** veil, swab, tampon, female genital secretions, proteins, DNA, HPV, release

## Abstract

**Background/Objectives**: Self-collection devices are more widely used than ever for detecting sexually transmitted infections and cervical cancer. Despite this, we still lack a clear understanding of how well these tools actually collect and release the necessary molecular samples. This study compared the in vitro uptake and release performance of commonly used self-sampling devices for total proteins, nucleic acids, and episomal human papillomavirus type 16 (HPV-16) DNA. **Methods:** An artificial cervicovaginal fluid composed of phosphate-buffered saline supplemented with serum and nucleic acid extracts was serially diluted 2-fold. Each dilution was applied for 5 min to the external surfaces of a vaginal veil (Vaginal Veil Collector V-Veil UP2^TM^ device), a flocked swab (FLOQSwabs^®^), and a commercial vaginal tampon. Non-woven surgical tissue and plastic film served as controls. Total proteins and nucleic acids were quantified by spectrophotometry, and HPV-16 DNA by real-time quantitative PCR. **Results:** Recovery rates for proteins and nucleic acids were highest for the vaginal veil (81% and 91%), followed by the swab (66% and 70%) and non-woven tissue (44% and 47%). In contrast, the tampon and plastic film performed poorly, releasing less than 30% of proteins and negligible amounts of nucleic acids. Episomal HPV-16 DNA release was highest for the veil (89%), compared with the swab (57%), non-woven tissue (37%), tampon (4%), and plastic film (2%). **Conclusions:** The vaginal veil demonstrated superior uptake and release of proteins, nucleic acids, and HPV-16 DNA at physiological concentrations. Its non-absorbent structure allows high saturation with efficient release of genital components, including microbial genomes, whereas vaginal tampons retained these components, limiting analytical recovery.

## 1. Introduction

Cervical cancer remains a significant global health burden. Yet participation in screening programs is frequently hindered by logistical, cultural, and psychological barriers. To address these challenges, the self-collection of genital samples has emerged as a transformative alternative that empowers “hard-to-reach” populations and supports screening in resource-constrained settings [1,2,3,4,5,6,7]. Different devices, including veil [8,9] and swab [10], as well as vaginal tampon [11], mainly in resource-limited settings, have been proposed for vaginal self-sampling [12,13]. While the clinical utility of self-sampling is well recognized, the diagnostic reliability of these programs is fundamentally tethered to the pre-analytical performance of the collection device. Thus, the efficacy of a self-sampling device—whether a vaginal veil, flocked swab, or tampon—is determined by its ability to both capture and subsequently yield molecular targets from female genital secretions, such as total proteins, nucleic acids, and oncogenic HPV DNA. This dual requirement of uptake and release depends fundamentally on the physical properties of the biomaterial used on the device’s surface.

Different biomaterials interact with cervicovaginal secretions through distinct physical mechanisms: (i) adsorption versus absorption: high-performance devices, such as the vaginal veil composed of non-woven hydrophilic polyethylene, are designed to adsorb secretions, allowing for a high saturation level and efficient desorption during the elution phase; (ii) capillary action: second-generation flocked swabs utilize perpendicular polymer fibers to capture liquid samples through capillary action, aiming for instantaneous elution; and (iii) sequestration: conversely, traditional absorbent materials like those found in vaginal tampons may act as sponges, absorbing and retaining various components, which can limit the analytical recovery of microbial genomes and lead to potential false-negative results.

There remains a lack of comparative data regarding how their specific physical properties of self-sampling devices—such as surface area and fiber composition—influence the recovery of soluble genital components. This study seeks to address this gap by evaluating the in vitro capabilities of three distinct self-collection vaginal devices to uptake and release the primary molecular components—specifically total proteins and nucleic acids—of female genital secretions. The differential molecular detection of HPV-16 DNA by the self-sampling devices was also assessed in vitro.

## 2. Materials and Methods

The evaluated self-sampling devices were the vaginal veil (Vaginal Veil Collector V-Veil UP2^TM^ device, V-Veil-Up Production SRL, Arges, Romania), the flocked swab (FLOQSwabs^®^, Copan Diagnostic Inc., Murrieta, CA, USA), and a commercial vaginal tampon (Tampons Sans Applicateur, Soft Mini, Carrefour France, Caen, France). Two controls were used, including commercial absorbent non-women tissue for surgery, and non-absorbent plastic film.

An artificial medium mimicking the cervicovaginal fluid was created with phosphate buffered saline (PBS) completed by serum and nucleic acids extracts, in order to reach the composition of normal female genital secretions at final proteins and nucleic acids concentration of 3035 µg/mL and 205 ng/µL, respectively. Commercially available episomal HPV-16 DNA (ACCURUN^®^ 378 SERIE 5000, SeraCare Life Sciences, Milford, MA, USA) was further added at constant level (3.17 log copies per 10,000 cells).

**Figure 1:**
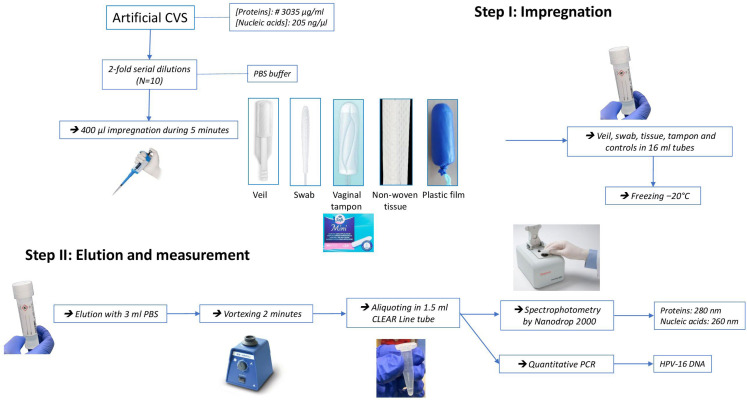
Flow chart of the experimentation evaluating in vitro the capabilities of three devices for self-collection of vaginal samples, including vaginal veil, flocked swab, and vaginal tampon to uptake and further release the principal components (e.g., total proteins and nucleic acids) of female genital secretions as well as episomal HPV-16 DNA. Absorbent non-women tissue and non-absorbent plastic film were used as controls.

Figure 1 depicts the flow chart of the experimentation. Ten serial 2-fold dilutions of artificial cervicovaginal medium were further carried out. For the collection step, 400 µL of each dilution were deposited for exactly 5 min on the external surfaces of the veil, flocked swab, vaginal tampon, non-woven tissue, and plastic film, and then kept frozen immediately at −20 °C.

For the elution step, the frozen impregnated devices and controls were eluted in 3 mL of PBS at room temperature, then vortexed during 2 min, and aliquoted in 1.5 mL tube.

The release of total proteins and that of total nucleic acids were finally measured by spectrophotometry using NanoDrop 2000/2000c (Thermo Fisher Scientific Inc., Waltham, MA, USA) at 280 and 260 nm, respectively. Cell-associated HPV-16 DNA was quantified using fluorescence-based Bioperfectus Multiplex Real Time Human Papillomavirus Genotyping Real Time PCR Kit with Perfectus HPV Analyser Software v1.0 (Jiangsu Bioperfectus Technologies Co., Ltd., Taizhou, China), as previously described [14,15].

## 3. Results

**Figure 2:**
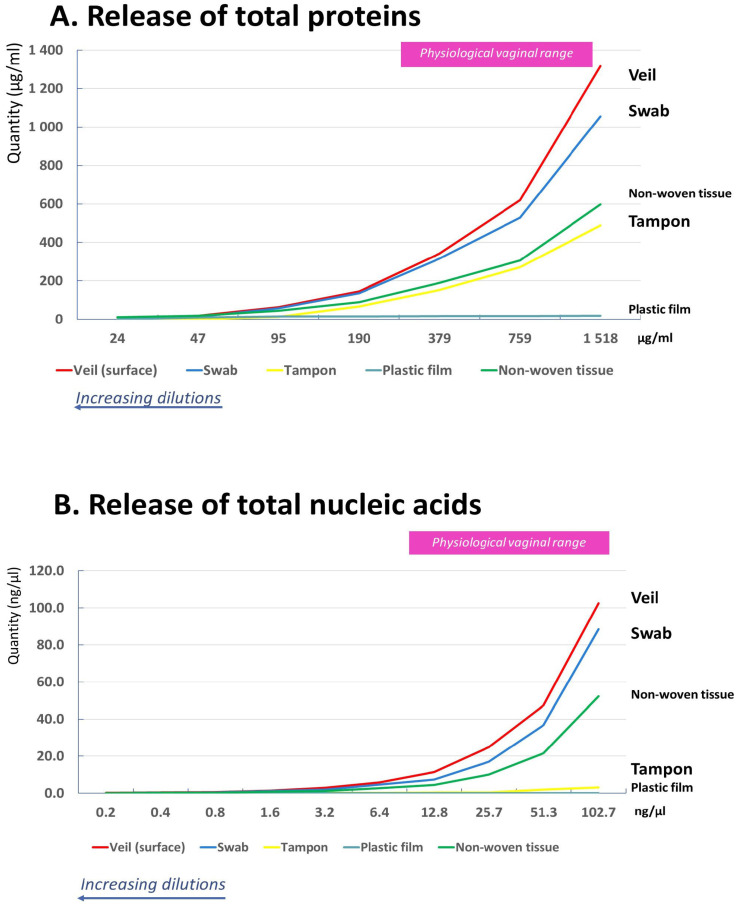
Release of total proteins in µg/mL (**A**) and total nucleic acids in ng/µL (**B**) at 2-fold serial dilutions in PBS of an artificial medium mimicking the human female genital secretions by veil, flocked swab, absorbent vaginal tampon, absorbent non-woven tissue, and non-absorbent inert plastic film.

The release of genital components from the external surface of the veil, the flocked swab, the vaginal tampon, the non-woven tissue, and the plastic film are shown in Figure 2.

The values obtained with the non-absorbent plastic film, as an inert surface, were considered as background noise for proteins and nucleic acids measurements.

**Figure 3:**
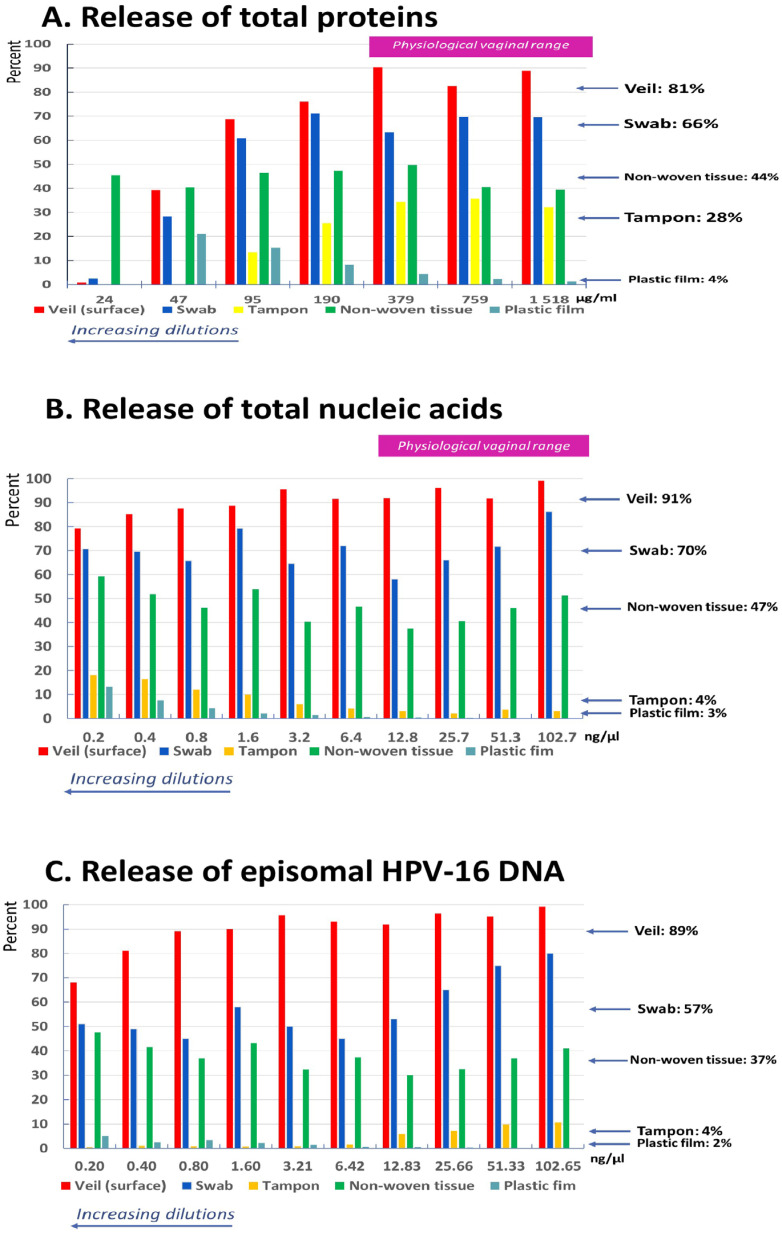
Release in percent of total proteins (**A**), total nucleic acids (**B**), and episomal HPV-16 DNA (**C**) at 2-fold serial dilutions in PBS of an artificial medium mimicking the human female genital secretions by veil, flocked swab, absorbent vaginal tampon, absorbent non-woven tissue, and non-absorbent inert plastic film.

The release of total proteins was maximal for the veil, followed by the swab, the non-woven tissue, and the vaginal tampon. In mean, the quantity of proteins release for the veil was 1.2-fold, 1.8-fold, 2.9-fold, and 12.9-fold higher, respectively, than for the swab, the absorbent non-woven tissue, the vaginal tampon, and the plastic film. The mean ± standard deviation (SD) of total proteins release was 81 ± 9.1% for the veil, 66.8 ± 6.4% for the swab, 44.6 ± 4.5% for the non-woven tissue, and 28.2 ± 9.1% for the vaginal tampon (Figure 3A).

The release of total nucleic acids was maximal for the veil, followed by the swab, the non-woven tissue, and the vaginal tampon. In mean, the quantity of nucleic acids release for the veil was 1.3-fold, 1.9-fold, 11.6-fold, and 30.2-fold higher, respectively, than for the swab, the absorbent non-woven tissue, the vaginal tampon, and the plastic film. The mean ± SD total nucleic acids release was 90.7 ± 5.8% for the veil, 70.3 ± 7.8% for the swab, 47.4 ± 6.8% for the non-woven tissue, and only 4.5 ± 2.6% for the vaginal tampon (Figure 3B).

The mean ± SD of episomal HPV-16 DNA release was 89.9 ± 9.1% for the veil, 57.1 ± 12.3% for the swab, 37.9 ± 5.4% for the non-woven tissue, and only 4.0 ± 3.9% for the vaginal tampon (Figure 3C).

The release of total proteins, that of total nucleic acids, and that of episomal HPV-16 DNA, were maximal for the veil at physiological vaginal concentrations.

## 4. Discussion

In the present study, the uptake and release of total proteins, total nucleic acids, and episomal HPV-16 DNA were maximal for the veil at physiological vaginal concentrations, followed by the flocked swab and, far behind, by the vaginal tampon. The non-woven tissue control showed middle properties of uptake and release, whereas the plastic film was not able to uptake and release significant amounts of vaginal components as well as HPV DNA. These observations demonstrate the differential physical properties of the external biomaterial used in self-sampling devices for the capture and release of vaginal components. The uptake and release of total proteins, nucleic acids, and episomal HPV-16 DNA are significantly influenced by the physical properties of the device’s biomaterial.

The vaginal veil demonstrated the highest capacity for both the acquisition and recovery of various biochemical components of female genital secretions, including episomal HPV-16 DNA. Furthermore, based on the study results, the vaginal veil’s high recovery performance was remarkably consistent across all ten serial dilutions. In Figure 3, the red bars representing the veil showed very low variability in percentage recovery for all three molecular targets: (i) total proteins: the veil maintained a high release percentage (averaging 81%) across the dilution range; (ii) total nucleic acids: the recovery was consistently high, with a mean of 91%; and (iii) HPV-16 DNA: the veil consistently released approximately 89% of the DNA, regardless of the concentration. This low variability across a 1024-fold concentration range (ten 2-fold dilutions) significantly strengthens the findings by indicating that the veil’s adsorbent efficiency appears independent of the solute concentration. It is likely that the special tissue of the veil allows high saturation level, with ad hoc release of genital components, including microbial genomes. Indeed, the vaginal veil is made of a hydrophilic non-woven polyethylene material [8]. The Vaginal Veil Collector V-Veil UP2^TM^ device catches and retains safely and gently the female genital secretions, thus harvesting cells, proteins, and nucleic acids (DNA/RNA). By its composition, the veil device does not absorb the liquids, but rather adsorbs the genital biochemical components, which allowed their full desorption after adequate elution of collected biological material, as previously reported for polyethylene non-woven polyesters [16]. The veil’s large external surface —contact surface: 6.75 cm^2^ [8]—and its particular biomaterial composition capable of adsorbing a maximum of proteins and nucleic acids and further efficiently releasing them from the impregnated veil according to the concentration gradient established during the elution phase, are what make the veil so original. The vaginal veil has been established as a user-friendly and powerful self-collection method, offering high acceptability for the molecular screening of oncogenic HPV [8,17]. Interestingly, two previously published studies comparing the efficiency of veil-based self-sampling to classical provider-based collection of genital secretions showed that vaginal specimens self-collected through the veil provided better detection of oncogenic HPV than collection by swab [8] or scraping [9]. Thus, in Chad, Nodjikouambaye et al. reported that veil-based genital self-collection was non-inferior to clinician-based collection as reference for HPV DNA molecular testing, with “good” agreement between the two collection methods, high sensitivity of 95.0%, and specificity of 88.2% [8]. Furthermore, positivity rates for any HPV DNA and high-risk HPV DNA were significantly higher with veil-based self-collection than with clinician-collected cervical swabs (1.67- and 1.57-fold increases, respectively; *p* < 0.0001) [8]. In Spain, the Vitroveil^®^ (VITROSA, Granada, Spain; similar to Vaginal Veil Collector V-Veil UP2™) self-sampling device allowed researchers to obtain a higher prevalence of molecular detection of any high risk-HPV than clinician-collected samples by cervical scraping (30.6% versus 24.3%; *p* < 0.0001) [9]. Our in vitro results demonstrate that the adsorbent properties of the vaginal veil optimize the capture-and-release cycle, which may explain its high sensitivity and superior detection rates of oncogenic HPV observed in previous clinical trials using conventional swabs or scrapings.

The flocked swab showed similarly high uptake and release of the proteins and nucleic acids of the female genital secretions as well as of the episomal HPV-16 DNA. FLOQSwabs^®^ belongs to the second generation of swabs with a flocking design, in which the swabbing head is comprised of polymer fibers that are linked to the shaft at a 90-degree angle [18]. The introduction of flocked swabs spiked their use in the medical field, mainly because they exhibit higher recoveries in specimen collection than conventional swab materials in the pre-analytical phase of microbiology [18,19,20,21]. The flocked swab comprises of a solid plastic applicator, which can be molded into various anatomical shapes, ensuring patient comfort without compromising the quality of the device. Glue is applied to the applicator tip and then tens of thousands of short nylon strands are sprayed (or flocked) onto the tip [18]. The perpendicular fibers adsorb liquid samples by the capillary action created by the surface tension between the nylon strands. Thus, flocked swabs release around 80% in volume of the nasopharyngeal secretions for influenza testing [18], demonstrating high capacities of uptake and release of mucosal secretions. According to the manufacturer, FLOQSwabs^®^ allows uptake and instantaneous elution of over 90% of the sample into the liquid medium [22]. While the swab’s design excels at releasing cellular material, our study, however, reveals that the veil may be superior for recovering soluble biomarkers, which could be highly relevant for further viral load and proteomic studies. Thus, in this experimental system and according to the conducted protocol, the uptake and release of biochemical components of the artificial genital fluid medium by the flocked swab showed relatively middle rates, of 66% for total proteins, 70% for total nucleic acids, and 57% for episomal HPV-16 DNA, which were less important than those of the veil. These findings could have two explanations. Firstly, the artificial medium mimicking normal cervicovaginal secretions used in our study was cell-free, and only soluble components, including proteins and nucleic acids, were taken into account and measured. However, the high capacity of uptake and release of the FLOQSwabs^®^ previously reported concerns the totality of mucous secretions [18], including cells, and not only the soluble components, as in our protocol. Second, it is likely that the smaller external surface area of the swab compared to that of the veil may explain at least in part the differences in uptake and release performances between the swab and the veil. Indeed, the estimated surface of FLOQSwabs^®^ is estimated at 3.24 cm^2^ (diameter, 5.5 mm and length, 16 mm) [23], thus 2-fold less than that of the veil. Finally, these in vitro observations on lesser efficiency of the flocked swab to uptake and release of soluble components of female genital secretions could also contribute to explain in part the higher diagnosis performance of the veil to detect oncogenic HPV by molecular biology by comparison with the flocked swab, as observed in vivo [8,9].

Lastly, the capabilities of uptake and release of female genital secretions components were much higher for the veil and the flocked swab than those of the vaginal tampon. The absorbency constitutes the crucial property of a vaginal tampon, referring to the tampon’s capacity to soak up and retain menstrual fluid [24,25]. The absorbency is related to the materials used (cotton, rayon, or blends), the density, and the physical dimensions as well the structural design of the tampon. Thus, the tampon behaves like a sponge, effectively absorbing genital secretions but failing to release the captured molecular components, with only middle or residual release of total proteins and nucleic acids, respectively. Otherwise, in laboratory practice, it is difficult to use impregnated vaginal tampons as samples. Indeed, because the components are trapped deep within the material, the restitution of genital secretions from an impregnated tampon requires either their compression, e.g., with a syringe, or a high quantity of elution liquid, with a high dilution of the secretions collected. These findings suggest that the vaginal tampon is an ineffective tool for cervicovaginal self-sampling, as it tends to retain rather than release captured molecular components, even if its cost is very low. Because of its low capacity to release nucleic acids and HPV genome, self-sampling with a vaginal tampon increases the risk of false-negative results in molecular screening.

New prevention strategies for the prevention of cervical carcinoma, particularly those using self-sampling of female genital secretions for HPV molecular testing, are particularly suited to resource-constrained settings for hard-to-reach populations or when medical facilities are lacking [6]. Self-sampling represents an impactful and important opportunity to support engagement in cervical screening to improve the participation of women in screening programs [12,13]. However, the steps required to support the end-to-end process are not trivial and require proper consideration and optimization to ensure maximum quality. Pre-analytical conditions of female genital secretions sampling constitutes the primary sine qua non condition of the possibility to optimize the analytical step of molecular detection of oncogenic HPV, in addition to correct collection, storage, and transportation of biomaterials to the laboratory [26,27]. Thus, assessing the operating properties of self-sampling collection devices is necessary to understand what problems may arise when the devices are used to obtain clinical samples for laboratory analysis. Our in vitro study, although reductionist, sets out the a priori optimal conditions of efficiency that devices for collecting cervicovaginal secretions must present, and shows that the physical properties of the surface biomaterials used in self-sampling devices are fundamental and discriminating.

Study limitations. Despite the significant insights gained regarding the material-dependent recovery of molecular targets, our study has several limitations that warrant consideration when translating these findings to clinical practice. Primarily, our study is obviously only in vitro. Although the episomal HPV-16 DNA used in the study could mimic cell-associated HPV, in real practice, the vaginal discharge from collection consists of vaginal epithelial cells and secretions. The use of a cell-free artificial medium, while necessary for standardization, does not fully replicate the complex rheological and biochemical environment of native cervicovaginal fluid. The absence of cervicovaginal mucus (mucins), shed epithelial cells, and microbial components—all of which are present in vivo—represents a critical gap. In a clinical setting, these elements significantly increase fluid viscosity and can lead to physical “clogging” of the device fibers or non-specific binding of DNA and proteins to cellular debris or the mucin network. Such interactions could potentially hinder elution efficiency or alter the saturation limits of the materials in ways not captured by our in vitro model. For example, the high viscosity of native mucus may impede the capillary action of the flocked swab or the surface adsorption of the vaginal veil more than the aqueous buffer used here. Consequently, while our results provide a clear baseline for the inherent performance of these biomaterials, the absolute recovery percentages in a clinical population may vary based on individual physiological differences in cervicovaginal secretions composition and the presence of vaginal dysbiosis. Finally, while this in vitro model is perfect for isolating and evaluating specific device-material properties, in vivo validation could constitute the essential next step, as the presence of mucus and high cellularity in native cervicovaginal fluid may increase viscosity and cause non-specific binding or physical entrapment.

In conclusion, our study, although limited, demonstrates that the physical properties of the biomaterial used to collect and release the various components of cervicovaginal secretions are decisive for sharing self-sampling collection devices. All these factors must now be taken into account in the development of self-sampling devices for primary molecular diagnosis of cervical oncogenic HPV.

## Data Availability

The data that support the findings of this study are available from the corresponding author upon reasonable request.
